# A Rare Case of Benign Esophageal Schwannoma

**DOI:** 10.7759/cureus.99852

**Published:** 2025-12-22

**Authors:** Abdullah Bahadi, Tehreemah Raziq, Thaabit Raziq, Hassan Robaidi, Wael Ahmed, Waleed Saleh

**Affiliations:** 1 Thoracic Surgery, King Faisal Specialist Hospital and Research Centre, Riyadh, SAU; 2 Medicine, Alfaisal University, Riyadh, SAU; 3 Lung Transplant, King Faisal Specialist Hospital and Research Centre, Riyadh, SAU

**Keywords:** benign schwannoma, esophageal nerve sheath tumor, esophageal schwannoma, esophagus schwannoma, schwannoma in a saudi patient

## Abstract

Esophageal schwannomas are rare, typically benign tumors arising from nerve sheath cells. Due to their low incidence, existing knowledge is largely based on individual case reports, limiting comprehensive understanding of their clinical behavior and optimal management. This case highlights diagnostic and surgical considerations in a 32-year-old male initially diagnosed with lymphoma. It is the first documented case in the country, further consolidating the rarity of the condition and illustrating diagnostic complexities through interdisciplinary management.

## Introduction

Esophageal schwannomas are rare, benign tumors originating from Schwann cells in the esophageal wall. They are more prevalent in females and typically affect individuals aged 45-70 years. Common symptoms include dysphagia, chest pain, dyspnea, and weight loss. Most tumors reside solitarily in the submucosa of the esophageal posterior wall, with the majority located in the upper or middle third [1,2].

Surgical resection is the cornerstone of treatment, although conservative management has been reported in a stable patient with intermittent dysphagia [3]. Other surgical approaches include thoracotomy, video-assisted thoracoscopic surgery (VATS), endoscopy, the cervical approach, and the Ivor-Lewis procedure. Post-operative recurrence is uncommon, with a single reported case of metastatic malignant schwannoma requiring further surgery [4]. Due to the rarity of evidence and most tumors being submucosal, biopsy, which is essential in diagnosis, has the potential to scar the area between the mucosa and muscularis. This could consequently make resection more difficult, requiring opening of the mucosa and eventually primary repair of the esophagus as opposed to a simple resection of the schwannoma.

Currently, there are no other published reports in the Kingdom of Saudi Arabia or the Gulf region. Due to the rarity of the condition, there are also no standardized management guidelines tailored to esophageal schwannomas in particular.

## Case presentation

A 32-year-old male mechanic, medically free with a smoking history of 16 pack-years, was referred from a local hospital with a mediastinal mass, initially suspected to be lymphoma. He reported experiencing difficulty swallowing both liquids and solids over the past three weeks. Despite the swallowing issues, there were no accompanying symptoms of weight loss, fever, or anorexia. The patient had no previous history of esophageal motility disorders or any instrumentation of the esophagus. At a primary healthcare facility, he was initially diagnosed with pharyngitis and treated with antibiotics, but symptoms persisted.

Upon physical examination, the patient appeared well, without any signs of respiratory distress or drooling. His vital signs were within normal limits, and his Eastern Cooperative Oncology Group (ECOG) score was 0.

**Figure 1:**
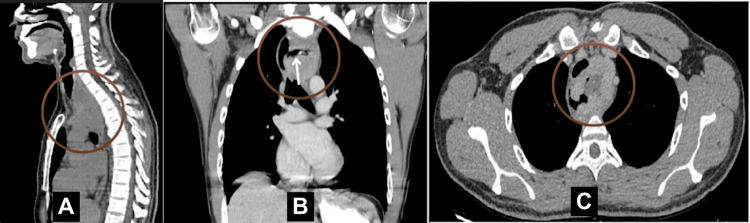
CT chest of the patient’s sagittal (A), frontal (B), and axial (C) views The brown circles in each view show the esophageal schwannoma, while the white arrow in the frontal view (B) denotes the fistulous tract extending from the esophageal lumen to the mass, which was not seen on EGD. EGD: esophagogastroduodenoscopy; CT: computed tomography

Initial investigations included a chest computed tomography (CT) scan that revealed an 8×6×3.5 cm mass in the superior middle mediastinum, invading the posterior esophageal wall (Figure 1). An abdominal CT scan further demonstrated numerous low-attenuation hepatic foci, too small to be fully characterized but likely representing cysts. The hepatic cysts were scattered and variable in size, with features suggestive of simple cysts or biliary hamartomas, and no malignant features were evident on magnetic resonance imaging (MRI). Moreover, contrast swallow revealed communication between the esophageal lumen and mass, possibly due to mass ulceration with fistulization.

Consequently, esophagogastroduodenoscopy (EGD) and endoscopic ultrasound (EUS) were arranged after a multidisciplinary discussion with interventional gastroenterology. EGD showed external compression of the esophagus with a large mucosal defect between 16 and 20 cm from the incisors, along with granulation tissue and necrotic changes. There was no evidence of neoplasia in the esophagus, and the stomach and duodenum appeared normal. Histopathological examination of the mucosal and fine-needle biopsies (FNB) revealed a spindle-cell neoplasm of nerve sheath origin, strongly favoring a schwannoma. Biopsies of the esophagus showed active granulation tissue, but no malignant features were present. Further immunohistochemistry tests were positive for S100 protein and SOX10 and negative for EMA and CD34, supporting the diagnosis of a benign schwannoma.

After these findings, the case was discussed at a sarcoma tumor board, where surgical excision with possible esophagectomy and pharyngo-gastric anastomosis at a more experienced center was recommended.

**Figure 2:**
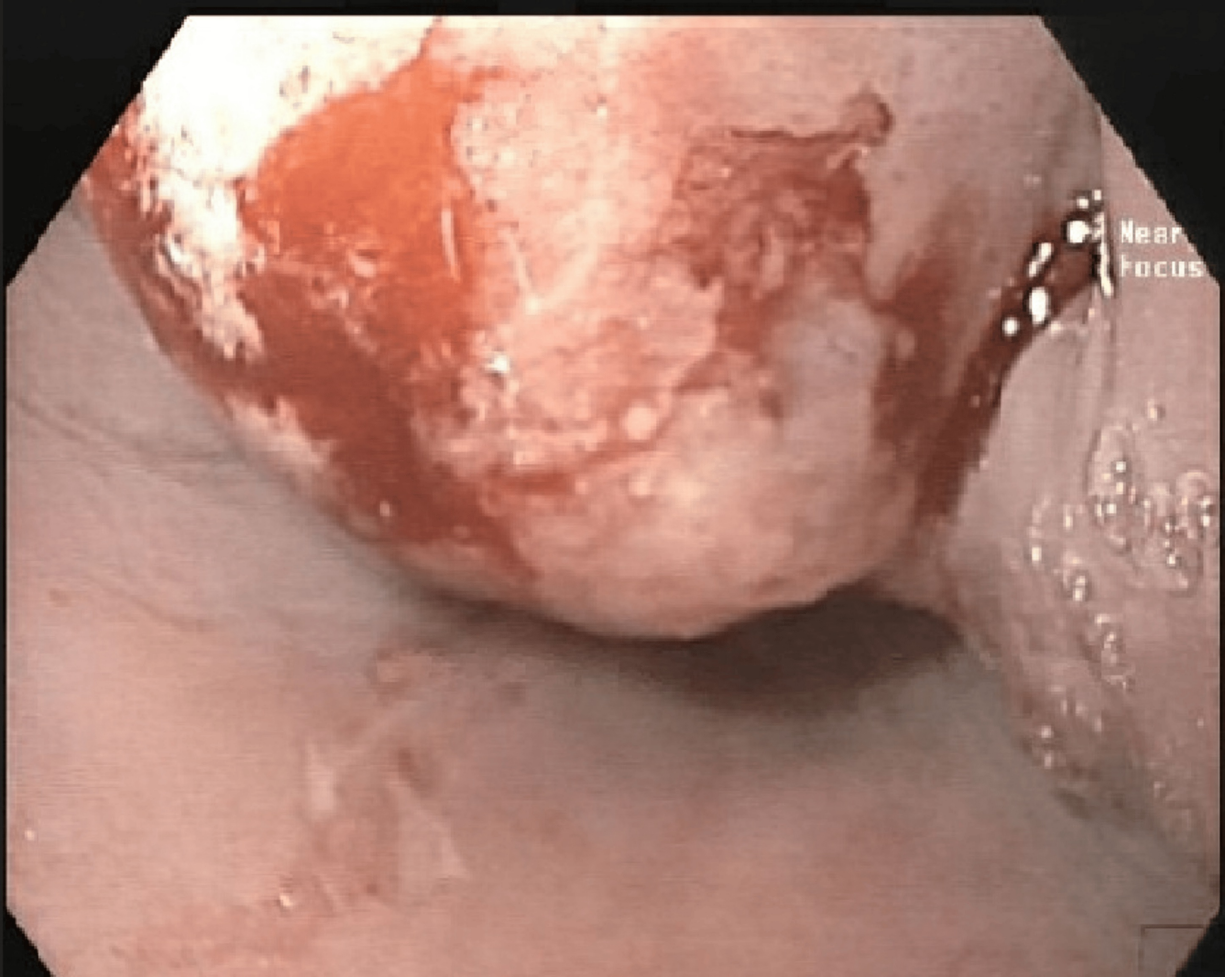
Endoscopic view of esophageal mass

Following his referral to our center, EGD was repeated, revealing a smooth intubation of the upper esophageal sphincter (UES). A mass was identified 20 cm from the incisors, approximately 2 cm below the UES, with a smooth surface and no nodularity, obstructing about one-third of the esophageal lumen (Figure 2) and no fistulas. Consequently, a discussion at our center's tumor board agreed with the surgical recommendation depending on intraoperative findings.

**Figure 3:**
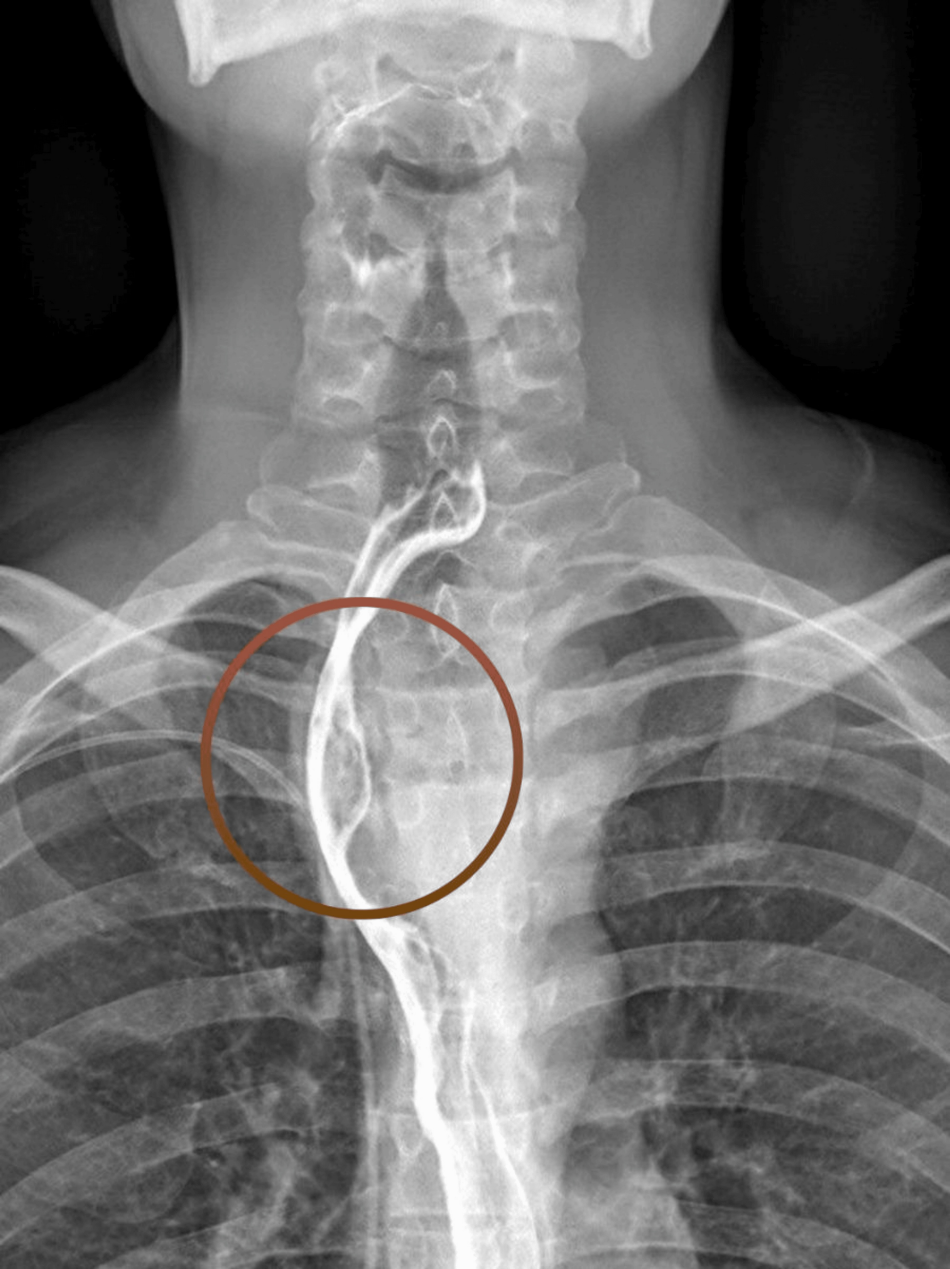
Contrast swallow fluoroscopy The esophageal mass can be seen externally compressing the esophageal lumen (brown circle). The fistulous tract between the mass and the lumen seen on CT cannot be appreciated here.

In preparation for surgery, a contrast swallow fluoroscopy study was performed, showing indentation of the cervical esophagus caused by mass effect (Figure 3). Through a cervical approach, a left-side neck incision was made anterior to the sternocleidomastoid, and dissection was carried out with preservation of the recurrent laryngeal nerve. The esophageal mass was well encapsulated but adherent to the wall at the level of the mucosa. The tumor was resected from the esophagus, resulting in opening the esophageal mucosa to achieve a complete gross margin, leading to an esophageal defect approximately 3 cm in diameter. A primary esophageal repair was performed using a single-layer closure with 3-0 PDS sutures, followed by a second reinforcing layer over the muscular portion. An interpositional flap was fashioned from a portion of the sternocleidomastoid muscle to provide additional support over the repair site. The closure was completed using 2-0 Vicryl sutures for the muscle and platysma, the skin was closed with staples, and drains were inserted.

The patient tolerated the procedure well with no intraoperative or postoperative complications. Consequently, he progressed in his diet and was discharged home on the fifth day in good condition. He was seen in the clinic three months after surgery with no active complaints. As the mass was proven to be benign and the patient had no postoperative complications, he was subsequently discharged from the clinic.

## Discussion

Esophageal schwannomas are rare neoplasms. Daimaru et al. identified only 24 gastrointestinal schwannomas among 306 spindle-cell tumors, highlighting their low incidence. These tumors typically show strong S-100 and SOX10 positivity, confirming Schwann cell origin, likely from the myenteric plexus [5]. They usually occur in women aged 50 years and older and involve the mid- to lower esophagus. Most patients present with dysphagia, and tumors commonly measure 4-8 cm [2]​​​​​. Our case, a 32-year-old man with an upper esophageal lesion, therefore represents an atypical presentation in age, sex, and location.

Distinguishing schwannomas from other submucosal esophageal tumors, particularly leiomyomas and gastrointestinal stromal tumors (GISTs), is challenging due to overlapping clinical and imaging features, yet crucial when considering organ-preserving management. In this case, the predominantly submucosal appearance closely mimicked a leiomyoma. Some authors advise against routine biopsy of suspected leiomyomas, as scarring between the mucosal and submucosal layers may complicate later dissection or enucleation.

Although most esophageal schwannomas are benign, malignant transformation has been reported [4,6]. Accurate histopathological and immunohistochemical assessment is therefore essential.

A tract between the mass and the esophageal lumen was identified radiologically. It is unclear whether this resulted from tumor-related ulceration or was iatrogenic following prior EUS and biopsy. No similar fistulization has been reported in the literature.

Surgical excision remains the mainstay of treatment, with approaches, including VATS, cervical incision, thoracotomy, and, in select cases, endoscopic resection, chosen based on tumor size and location [4,7,8].

This case adds to the limited global experience with esophageal schwannomas and, to our knowledge, is the first reported from Saudi Arabia, contributing to the broader understanding of their clinical behavior and management.

## Conclusions

This case describes a 32-year-old medically free male diagnosed with a benign esophageal schwannoma in the mucosa of his upper thoracic esophagus. Due to the size of the mass, histological location, and suspicion of a fistulous tract before surgery, the tumor was resected and followed by primary esophageal repair via a cervical incision. There were no complications, and consequently, the patient made an uneventful return to baseline.

Furthermore, although esophageal schwannomas have been previously reported, the number of documented cases remains exceedingly scarce worldwide. Given this limited global experience, each additional well-characterized case contributes meaningfully to the existing body of literature. Reporting our case not only reinforces the clinical spectrum and management considerations of this uncommon tumor but also adds valuable data that may support future systematic reviews. Such aggregated evidence will be essential for improving understanding of the disease's behavior, refining diagnostic pathways, and guiding optimal surgical management.
